# Rare diseases, body, and stigma: a comparative analysis of people living with Berardinelli Syndrome and Machado–Joseph Disease in Brazil

**DOI:** 10.1007/s12687-026-00950-3

**Published:** 2026-10-08

**Authors:** Waleska Aureliano, Carlos Guilherme Octaviano do Valle, Jociara Nóbrega

**Affiliations:** 1 Department of Anthropology, State University of Rio de Janeiro, Rio de Janeiro, RJ Brazil; 2 Department of Anthropology, Federal University of Rio Grande do Norte, Natal, RN Brazil; 3 Department of Education, Government of the State of Paraíba, São Jose do Sabugi, PB Brazil

**Keywords:** Stigma, Rare diseases, Berardinelli Syndrome, Machado-Joseph Disease

## Abstract

**Objectives:**

This article analyses how people living with rare diseases are stigmatised due to physical features and bodily alterations, considering the moral dimensions through which these differences are read. The analysis draws on research conducted in two regions of Brazil (Northeast and Southeast) with people affected by two hereditary rare conditions: Berardinelli Syndrome (BS) and Machado-Joseph Disease (MJD). We selected these conditions because of their significant bodily transformations: BS is present from birth, whereas MJD emerges in adulthood. This enabled us to examine how stigma is managed within families across the life course and identify who, beyond those directly affected, experiences its impacts.

**Methods:**

The article draws on data from two research projects conducted at different times. Both involved ethnographic fieldwork in hospitals and public events organised by patient associations, as well as in-depth interviews with patients and family members. Materials were analysed through narrative analysis.

**Results and discussion:**

Our analysis was guided by three lines of inquiry concerning the construction and perception of stigma among people living with BS and MJD: (1) recognition of the first symptoms and lay interpretations of illness, leading to encounters with genetic explanations for its causation; (2) experiences of stigma through the interplay between bodily changes and gender expectations in the context of rare disease; and (3) health activism as a mechanism for coping with stigma.

**Findings:**

Our findings reveal how the intersection of body, stigma, and identity produces moral readings of people living with rare diseases, based on the social interpretation of bodily changes brought about by these conditions.

## Introduction

The World Health Organisation defines a disease as ‘rare’ when it affects fewer than 5 in 10,000 individuals. In Brazil, the threshold used is 65 in 100,000. Taken as a whole, rare diseases (RDs) affect between 3.5% and 5.9% of the global population. In Brazil, somewhere between 13 and 15 million people are estimated to live with some kind of rare disease. Approximately 72% of rare diseases have a genetic origin, whether hereditary or not, and many result in some form of physical and/or intellectual disability. Such physical and/or cognitive alterations may become grounds for stigmatisation when they are socially interpreted against expectations of bodily and cognitive normality. This may affect not only individuals living with the condition but also their families and descendants, particularly in the case of hereditary rare diseases (HRDs).

In this article, we analyse how the physical features and bodily alterations of people living with rare diseases are stigmatised, considering the moral dimensions through which these differences/alterations are socially interpreted. In our analysis of stigma, we considered dimensions of participants’ familial and community experiences, including perceptions that there was “something wrong with that family,” as well as individual experiences shaped by the social and cultural expectations of gender that may be disrupted by rare disease. From this perspective, we examine how the intersection of embodiment, gender/sexuality, and identity within the social and cultural imagination produces stigmatising moral interpretations of people living with rare diseases. We therefore consider how the meanings attributed to disease-related bodily transformations and forms of physical difference contribute to particular modes of embodiment and personhood through which affected individuals are socially recognised, classified, and understood.

In our reflection, the analysis of the category of social stigma draws from the social sciences in general, taking Erving Goffman’s work (1988) as its primary reference, but also centering on the debates and uses of the concept within the field of social and anthropological studies on health (Castro and Farmer 2005; Parker 2012; Scambler 2004, 2009; Hansen et al. 2014; Thomas et al. 2025). The discussions on body–health–illness that run through this field of studies are inseparable from the category of stigma and its frequently association with the concept of identity, including sexual and gender identities.

We draw on the recent analysis of Thomas et al. (2025), which highlights both the analytical potential of the concept of stigma and the pitfalls associated with its use in the social sciences. According to the authors, stigma has increasingly been employed as a broad and insufficiently specified explanatory category, often treated as a self-evident feature automatically associated with particular conditions, such as illness, disability, sexuality, or poverty, without adequate investigation into how it is produced in specific social contexts. This naturalised use risks transforming stigma into a “black box”, obscuring the complexity of social experience and limiting its explanatory value in health research.

Thomas et al. (2025) argue that Goffman’s central proposition was that stigma should be understood as a relational and interactional phenomenon, emerging in social situations where normative expectations are disrupted. From this perspective, stigma is not an inherent attribute of individuals but rather a process enacted through social interactions, in which the positions of the “normal” and the “stigmatised” remain fluid and contextually situated. The authors contend, however, that the literature—including work within the social sciences—has frequently misinterpreted this approach by treating stigma as a fixed and universal attribute. Such interpretations reduce the complexity of Goffman’s formulation and reinforce essentialising accounts of social difference. Building on this critique, Thomas et al. (2025) review contemporary contributions that seek to shift the analytical focus from stigma as an interactional phenomenon to the structural dimensions through which stigma is produced and sustained.

Scholars such as Scambler (2004, 2009), Tyler (2020), and Link and Phelan (2014) have emphasised that stigma is deeply embedded in relations of power, inequality, and political processes, operating as a mechanism of social exclusion. Within the field of health, this perspective requires attention to the role of institutions, public policies, and biomedical discourses in the production and reproduction of stigma, moving analysis beyond individual experiences towards the broader contexts that shape access to care and the distribution of resources. Analyses of stigma therefore need to engage both micro- and macro-sociological scales: the former illuminates lived experiences and everyday interactions, while the latter draws attention to the structures of power, inequality, and ideology that shape those experiences. In this sense, stigma may be understood as a simultaneously symbolic and structural phenomenon, manifesting both in everyday interactions and in institutional and political dynamics (Thomas et al. 2025). This perspective is particularly useful for our analysis, as it highlights how, in Brazil, people living with rare diseases have found in health activism an important locus for challenging the stigmas they encounter both in everyday social interactions and in the development of public policies addressing rare diseases (Aureliano and Gibbon 2020; Gibbon and Aureliano 2018).

Of course, not all individuals who suffer from a particular disease experience the stigmas associated with it or engage in a political movement. On the contrary, many internalize stigma because they are subjected to oppressive social structures and symbolic apparatus whose function is to legitimize inequalities based on hierarchical understandings of social value and normality. Nevertheless, it is essential to pay attention to how stigma is negotiated both at the level of micro-relations and in broader arenas of social action.

Our analysis is grounded in research conducted in two different regions of Brazil (the Northeast and the Southeast) with individuals affected by two hereditary rare conditions: Berardinelli Syndrome (BS) and Machado-Joseph Disease (MJD).

BS is a rare autosomal recessive condition with a high incidence of consanguinity (Berardinelli 1954; Sarmento et al. 2019). It is characterised as a congenital generalised lipodystrophy,^1^ which provokes an acute absence of body fat and an increased visibility of the musculature. BS produces bodily modifications typically identified in childhood or even soon after birth, the main feature being a reduction or absence of subcutaneous fat. Since the syndrome causes a variety of organic complications that may restrict physical and social functioning, in particular diabetes, liver problems and cardiac issues (Van Maldergem 2003), the biological pathologies associated with BS lead to strong involvement of family members in the search for treatments and an improved quality of life for their affected relatives or their descendants. In many cases, women affected by the syndrome have a body type that provokes question about their gender identity and sexual orientation, which can lead strangers to perceive them as ‘masculine’ – that is, as women who deviate from the gender norms socially expected of them. For men with the syndrome, by contrast, the increased muscle mass tends to reinforce ideal models of masculinity, generally affording them greater social acceptance.

An autosomal dominant inherited condition, MJD is one of the hereditary forms of ataxia. Though first identified in the medical literature in the 1970s, its genetic basis was only specified in the 1990s. It is the most common form of hereditary ataxia in Brazil (Jardim et al. 2001; Lopes-Cendes et al. 1997). The disease is late onset, generally appearing after the age of 40, and there is currently no cure or treatment. It affects the person’s balance, speech, vision and, at more advanced stages, swallowing and other motor functions. Symptoms such as loss of balance when walking and slurred speech frequently cause affected individuals to be perceived as intoxicated, in turn leading to intense stigma, a phenomenon observed in other research contexts on the disease (Couto et al. 2024; Paúl et al. 1999). This stigmatisation has a moral impact on both men and women, although the former tend to be more heavily affected in workplace environments.

The article is organised in two parts. In the following section, we present the methodology employed in our research, which combined long-term ethnographic fieldwork for data collection with other methods such as in-depth interviews. In the second section we present the results and discussions of the empirical material collected from our research with Brazilian families affected by BS and MJD, structured around three lines of analysis involving perceptions of stigma and the production of identities among people living with hereditary rare diseases: (1) the recognition of the first symptoms and lay interpretations of the illness, leading to the encounter with genetic explanations for its causation; (2) the experience of stigma through the interplay between bodily changes and gender expectations in the context of rare disease; and (3) health activism as a mechanism for coping with stigma.

We compared the experiences of people living with these two rare diseases because both entail significant bodily transformations, one present from birth and the other emerging in early adulthood. This enabled us to explore how stigma is negotiated within families across different stages of the life course and how its effects extend beyond the affected individual to shape the experiences of other family members.

## Methods

Both studies, qualitative and ethnographic in nature, were based on fieldwork carried out in hospitals, family homes, patient associations, and in-depth interviews with people living with BS and MJD, both symptomatic and asymptomatic, and receiving care through Brazil’s public and private healthcare systems, as well as with their family members, healthcare professionals and public officials.

Waleska Aureliano conducted research on the topic of rare diseases between 2013 and 2022, primarily in the city of Rio de Janeiro, the second most populous state capital in Brazil, located in the Southeast region. Although her research has examined rare diseases more broadly (Aureliano 2018; Aureliano and Gibbon 2020), in this article we present a specific case study focusing on families affected by Machado-Joseph Disease (MJD) (Aureliano 2015). The study involved fieldwork conducted between 2013 and 2014, initiated through contact with an association for individuals and families living with acquired and hereditary ataxias in the state of Rio de Janeiro, Ataxia-Rio, that was composed primarily of people from working-class backgrounds. The association was founded in 2012 by patients receiving care at a public hospital in Rio de Janeiro city where the author also conducted fieldwork, accompanying consultations in the institution’s medical genetics outpatient clinic during six months. The research consisted mainly of attending the association’s monthly meetings over a two-year period, with a total of 16 meetings observed. In addition, events in which association members participated were also observed, including three meetings with government officials engaging with the rare disease agenda in Rio de Janeiro. It was also possible to accompany medical consultations of some of the association’s members at the hospital where the fieldwork was conducted over the same period. This immersion enabled collection of most of the analytical data, focusing especially on the observation of spontaneous social interactions and the recording of the various topics that emerged in these encounters. These conversations almost always revolved around the rights of people with rare diseases, the challenges faced in everyday life (including ways of coping with disease-related stigma) and perceptions of risk and inheritance in the construction of family reproductive projects. Finally, ten formal interviews were conducted, seven with symptomatic and asymptomatic members of two families affected by MJD who attended the association frequently and had members among its founders, and three with healthcare professionals (two geneticists and one psychologist).

The research by Jociara Nóbrega and Carlos Guilherme do Valle was conducted in the Sertão do Seridó, an inland region situated between the states of Rio Grande do Norte and Paraíba in Brazil’s Northeast (Nóbrega 2020; Nóbrega and Valle 2023). The region is known for its high concentration of individuals living with Berardinelli Syndrome (BS). In Brazil, cases of the syndrome have been recorded in the states of Rio Grande do Norte, Paraíba, Ceará, Pernambuco, Minas Gerais, Rio de Janeiro and São Paulo (Sarmento et al. 2019). The research was conducted between 2015 and 2019, initially through contacts established with a patient and family association, the Association of Parents and People with Berardinelli Syndrome of Rio Grande do Norte (ASPOSBERN), founded in 1998. This association involved around thirty-seven families. Although the association’s main leaders come from middle-class backgrounds, the vast majority of participants have a rural and working-class socioeconomic profile, living in small towns and rural settlements in the Sertão do Seridó. Fieldwork consisted of observing the association’s annual collective meetings, three in total, as well as observing an event held at the Federal University of Rio Grande do Norte, which included the participation of ASPOSBERN’s leaders. In addition to observing these events, twenty-one formal interviews were conducted with people with BS, their parents, healthcare professionals, and public officials.

In both studies, data collection and analysis were conducted exclusively by the authors of this article, in accordance with the authorial nature of research in social anthropology. The people who took part in the studies were either affiliated with the patient associations cited above or, in the case of the health professionals we interviewed, maintained care relationships with them. During the research period, the position attributed to us within the associations was one of collaborators, since the research we were developing was seen by participants as an important contribution to increasing the visibility of rare diseases in Brazil and highlighting the demands of those affected by them. In the hospital setting, the first author’s participation was formally approved by the relevant institutions. The researcher’s presence during consultations and other activities conducted in these environments was subject to patients’ informed consent. Patients were also informed that medical confidentiality was ensured through anonymity and the non-disclosure of individual patient data in any published results from the research.

For the in-depth interviews, we selected individuals who held leadership roles in their associations, as well as patients and family members who regularly or sporadically attended the association’s meetings, whether symptomatic or not. Each study used a specific topic guide, adapted to the particular characteristics of each research field and the profile of each rare disease. Both sets of interviews had a semi-structured format, which allowed for the emergence of themes not initially included in the topic guides. The two guides were organized around some common axes: understanding how the genetic heritability of the rare disease was perceived by our interlocutors^2^; the impacts that this genetic knowledge had on the reproductive projects of the affected families and individuals; the pathways leading to diagnosis and treatment; the difficulties faced by affected individuals and families in their daily lives; and the role of associations in their trajectories.

We initially analysed the data from each study separately, given that the research was conducted at different times and in different settings. Through repeated readings of the interviews and fieldnotes, we identified recurring themes and contrasts within and across the two research contexts. Two themes were particularly recurrent across both bodies of material. The first concerned the search for a diagnosis within families and participants’ interpretations of illness before and after learning about its genetic cause and mode of inheritance. The second concerned the role of patient associations as spaces for personal and collective coping with illness and stigma, as well as for claiming rights. A third analytical theme emerged from our attention to participants’ accounts of bodily changes and gender roles, which offered a further perspective for understanding how stigma was experienced and interpreted in each condition. We therefore returned to the field materials to identify and select narratives that most explicitly articulated these three themes. This process was interpretive and iterative rather than based on formal coding, drawing on the meanings, connections, and contrasts that emerged through successive readings of the ethnographic materials and participants’ narratives.

The analysis drew on different forms of narrative material generated through ethnographic fieldwork, including in-depth interviews, illness accounts shared in public settings, and fieldnotes. It is important to emphasise that narrative should not be conflated with the interview itself; rather, the interview constitutes only one of several possible contexts for narrative production. Within this framework, narrative is understood as a broader interpretive construction that may emerge across different interactional contexts and that involves the selection, ordering, and attribution of meaning to lived events, taking into consideration their temporal, relational and cultural dimensions (Kleinman 1988; Good 1994; Mattingly 1998). This means that narratives may be understood as situated social practices, shaped by markers such as gender, sexuality, class, ethnicity/race, age, and articulated within broader institutional, political, and moral contexts. Consequently, the analytical focus rests not only on what is said, but also on how, why, and under what conditions something is narrated.

Finally, narratives do not concern only past events recounted in the present; they also emerge through social interactions and collective spaces, structuring the social action of those involved (Mattingly 2000). With this in mind, narratives should not be analysed as repositories of absolute truth, but rather as part of a continuous and fluid process of interpretation in which perspectives are taken up by actors and negotiated within social interactions (Riessman 1993).

Both studies were approved by the Ethics Committees for Research with Human Subjects of the institutions to which the authors are affiliated, as well as by the healthcare institutions involved^3^. All participants signed the informed consent form.

## Results and discussion

Since the present work involves two anthropological studies based on ethnographic research, we present the results and discussion simultaneously, recognising that the ethnographic text does not presume an analytical construction separate from the description of data. We prioritise exploring the data found in our research in the discussion, emphasising similarities with other scenarios when relevant, but above all, valuing the narrative of our interlocutors in the analysis.

This section is organised around three analytical axes through which we examine the stigma experienced by individuals and families affected by BD and MJD in Brazil, as mention above. We selected these three axes because they emerged as particularly salient across the narratives we collected, especially in observations conducted during patient association meetings and in-depth interviews.

To preserve the anonymity of research participants, we use the following descriptors: MJD or BS followed by a number to indicate participants with a formal diagnosis; AR followed by a number to indicate asymptomatic relatives who are at risk of developing the condition; and BSF followed by a number to indicate family members of people diagnosed with BS who are not at risk of developing the condition.

### ‘It’s in the blood’: first symptoms and the search for the origin of the ‘genetic inheritance’ in the family

It might be imagined that people affected by a hereditary rare disease (HRD) already have some prior knowledge of the condition affecting them due to experience of the illness in their family. However, this was not what we observed over the course of our studies. The search for the origin of the ‘genetic inheritance’ was a recurrent theme among the families we researched.

In the case of MJD, the grandparents and parents of the interviewees had passed away without ever knowing their actual medical condition, a common situation in the narratives of others who frequented the association. It was only after a correct diagnosis was made in individuals from the third generation that some of the questions regarding their grandparents and parents began to be cleared up. A significant point that emerged in the majority of accounts was the feeling of inadequacy experienced by these relatives who, undiagnosed, were left coping with the stigmas associated with the symptoms of the disease in public spaces:

*‘My father suffered a lot because nobody knew what it was; they [the doctors] were lost too. He faced prejudice, even from us, because we didn’t know what it was and thought he was just being dramatic, we started thinking he was drunk, you know? He went through a lot, because he suffered prejudice from outsiders and from those close to him, because even we, his own family, didn’t know what was happening’ (MJD-1, woman, 46 years old).*

*‘My father had [the disease], as did my aunt, his uncle and his brother too. But at that time, nothing was known about the disease […] the doctor would say to my father, ‘you have a degenerative disease with no cure’, but he wouldn’t name what it was and my father couldn’t deal with that, when the doctor said there was no cure, he became disheartened’ (MJD-2, man, 52 years old).*

Initiated with the recollection of the life of parents, this search often extends to older relatives, such as grandparents who ‘died paralytic,’ with even less comprehension of the illness that affected them, their symptoms frequently attributed to the ‘natural’ processes of ageing. The greatest challenge of living without a clear diagnosis was highlighted by those whose fathers began showing symptoms while they were still of working age. The lack of an explanation for the genetic basis of their condition, combined with the social stigma associated with disability, pushed these men into depression, causing them to ‘give in to the disease’:

*‘My father died at 73, but it was because he gave up. He heard the doctor say there was no cure, and after he stopped working, he just sat in a chair and stayed there until he died. He didn’t engage in any physical activity, he refused to walk with a cane, he even refused to use a wheelchair, he refused everything’ (MJD-2, man, 52 years old).*

*‘Our neighbours thought my father was an alcoholic because when he walked up the street, he would stagger from side to side. But we didn’t know, it was the disease’ (AR-1, woman, 62 years old).*

Even among those with a genetic diagnoses, some continued to adhere to the narrative passed down by their parents to explain the symptoms. One such case was a 73-year-old man with several relatives on his paternal side affected by MJD, who explained his late father’s use of a wheelchair as the result of an accident rather than the disease. Another man believed that his father’s condition had worsened not because of its degenerative nature, but because of a lack of physical activity.

The idea that it was possible to inherit the ‘defective’ gene without manifesting the disease, or manifest it in a ‘mild form’ only, was also common among the individuals we interviewed. What was considered a ‘mild’ manifestation referred mainly to the ability to walk steadily and have less impaired speech. When these two capacities were affected, they evoked the physical stigmas associated with disability and bodily incapacity, as well as the perception that the person might be intoxicated, a form of stigma that Goffman (1988) called a perceived ‘character weakness,’ associated, among other things, with personal vices.

The search for the origin of the disease within the family, the perception of the stigmas experienced by family predecessors in an era when diagnoses were limited, and the suffering of relatives who had ‘given in to the disease’ were all cited as elements that led interlocutors to reflect on their kinship relations and to seek alternative and more positive ways of living with their condition. Maintaining a healthy diet, exercising and ‘taking care of the mind’ were listed as strategies to prevent the disease and its symptoms from ‘progressing too quickly.’ In comparing their own experiences with those of their parents or grandparents, all the interlocutors felt they were in a better position than their predecessors since they now had ‘knowledge’ of the disease. This allowed them to negotiate their identities in public spaces as ‘rare disease patients’ rather than as ‘drunks,’ ‘paralytics’ or ‘disabled people.’

Along these lines, the category of ‘disabled person’ was seldom if ever invoked by the participants in our research, who did not identify themselves in this way, but rather as ‘a person with a rare disease.’ This choice can be understood as a strategy for navigating social stigma by adopting an identity category, someone with a *rare disease*, that is less marked by the prejudices frequently directed at people with disabilities. Even so, at the level of social interactions, the symptoms presented by people with MJD – such as the need to use a wheelchair, a walker frame or crutches, or their impaired speech – continue to be perceived as signs of disability.

In the case of people living with BS, the condition was historically known in the interior of the Rio Grande do Norte state as the ‘disease of the thin’ (*doença dos magros*) due to the pronounced thinness of those affected. This popular/lay term was used to refer to people affected by the syndrome until the end of the twentieth century, including by local health professionals. The disease was easily recognised due to its relatively frequent occurrence in the region, a fact associated with marriages between cousins or other close relatives, a practice common to the area since the nineteenth century (Dantas 2005)

To take one example, a mother of three girls with BS initially sought the opinion of various doctors in her region to deal with specific health issues such as dermatitis, itching and low weight. Long before seeking a formal diagnosis, this mother already ‘knew’ that her daughters had the *doença dos magros*, which had manifested in relatives from earlier generations and had affected other people in her local area. She recognised a kinship relation with some of these individuals – either close or more distant – meaning that the condition was understood to some extent as a ‘family disease’ (Nóbrega 2020). Similarly, the mother of a boy who exhibited accelerated growth and extreme thinness was asked by her son’s godmother whether the child might be ‘one of the thin,’ echoing this cultural interpretation of the condition. The child’s great-aunt, who had known a relative with the same characteristics, made the similar comparison: *‘Back then it wasn’t called a syndrome! They just said ‘the thin ones’’ (BSF-1).* Thus, the ‘disease of the thin’ can be understood as an emic category that, while permitting local identification and recognition of affected individuals, also produces stigma.

The medical explanation of the condition as Berardinelli Syndrome only became more widely known to the regional population in the twenty-first century due to the work of the local association of people living with the syndrome and their families. The identification of its genetic cause and the use of a medical nomenclature enabled both formal diagnosis and a reformulation of identity for those affected and their relatives. The popular and historical idea of the *doença dos magros* thus began to be contrasted with the biomedical language and explanatory models, the condition now explained as a syndrome provoking ‘congenital generalised lipodystrophy.’

For the people with BS in our studies, identifying the cause of the disease, its genetic origin, and learning the associated nomenclature not only afforded access to a high-cost treatment (leptin^4^), but also brought socio-relational and emotional benefits, as the stigmatising and pejorative regional label, ‘disease of the thin,’ gave way to the clinical identity of a ‘person with Berardinelli Syndrome.’

In both cases, we observed that knowledge about the rare disease in the family passed through two distinct phases. The first was related to the collective experiences of the families with their affected members, through which they recognised that there was “a disease in/of the family,” even though its aetiology remained unknown. This lack of knowledge shaped how social stigma was produced, perceived and experienced by the affected families, who at that moment had few explanatory tools about the condition to present in social interactions. In a second phase, families gained access to medical and scientific knowledge that explained the genetic origin of the disease and gave it a name. In both groups, we perceived that naming the disease was important to giving new meaning to the family’s history, obtaining access to more effective care, and activating less solitary forms of identification by enabling the recognition of other people affected by the same rare disease, particularly through the patient associations. Similar processes have been documented in other settings, where developments in genetic knowledge have facilitated the emergence of new clinical identities among families affected by rare diseases and have also stimulated forms of health activism through which stigma is contested and claims to health-related rights are advanced (Akrich et al. 2013; Rabeharisoa et al. 2012, 2013; Rose 2007; Novas 2006; Rabinow 1999), as we discuss below.

### Between being and seeming: gender expectations and stigma

As observed earlier, the unsteady walk and slurred speech of some patients with MJD are symptoms that often cause others to presume they are intoxicated. Narratives of social situations in which our interlocutors were mistaken for being drunk were frequently shared during the association’s meetings:

*‘The other day I went to fill up and when I got out of the car to pay with my card, the guy looked at me and said, ‘This old guy’s already drunk first thing in the morning’’ (MJD-3, man, 73 years old).*

*‘I was on my way to work when a woman stopped me in the street to hand me a church leaflet, saying that Jesus could cure me of my addiction and asked me what I’d consumed to be in that state so early in the morning! I couldn’t believe it, I was left speechless’ (MJD-4, woman, 30 years old).*

These accounts were so common that one of the association’s first initiatives was to design a T-shirt reading, ‘I’m not drunk, I have ataxia.’ The shirt was made by one of the families belonging to the association and was worn at meetings. After a while, though, they decided to remove the phrase referring to drunkenness. Some people felt it could reinforce rather than lessen the stigma, since the symptoms of MJD continued to be associated with alcoholism. It also risked stigmatising people with an alcohol problem who, after all, have medical issues too.

The association made between alcoholism and MJD symptoms, observed in other studies on ataxias (Couto et al. 2024; Paúl et al. 1999), was stressed by both men and women, albeit more emphatically by the former, especially when this confusion surfaced in the workplace. While the women who were working at the time of their diagnosis cited more subtle remarks from colleagues suggesting that they were ‘walking awkwardly’ or were ‘slower’ when performing their tasks, leading the women to suspect something was wrong, the men expressed from the outset the fear of being perceived as drunk by their co-workers. The memory of similar stigma experienced by their fathers, combined with the fact that men tend to consume alcohol more regularly, help explain this concern. Two of our interlocutors, both army officers, only spoke openly about the disease after genetic tests confirmed the medical condition. Without firm proof, they feared their explanations for underperformance at work would be dismissed by their peers.

*‘I began noticing the symptoms in 2003, 2004, but I kept going about my life at the barracks […] But by 2009 it had become difficult, because I could tell that people were noticing. […] There was a sergeant who worked with me and he told me he’d noticed. He said to me: ‘Major, you show up here early in the morning looking like you’re drunk.’’ (MJD-2, man, 52 years old).*

*‘I had a problem, whenever there was a [military] assembly, I would leave because I couldn’t stand still. You have to remain motionless, but I couldn’t, because my balance wouldn’t allow it. But I never said anything to anyone’ (MJD-3, man, 73 years old).*

In discussing information control and stigma, Goffman (1988) distinguishes between “discredited” individuals, whose stigmatising attributes are visible, and the “discreditable”, whose difference can be concealed. In the examples above, while the physical impairment caused by the disease remained subtle and no diagnosis had yet been disclosed, both individuals could be considered “discreditable”: there was a suspicion that something was wrong, but no certainty, and it was still possible to manage the visibility of symptoms in social interactions. Once the diagnosis had been confirmed, however, they came to see themselves—and to be seen by others—as “discredited” in relation to their professional roles, a process that ultimately resulted in the compulsory retirement of both individuals.

A contrasting response emerged in the case of a woman presenting the same symptoms. Her colleagues were understanding and even expressed disbelief that she could have the disease, despite the fact that her sister had already received the same diagnosis. The explanation ventured by the woman’s coworkers for her difficulties at work was that she was ‘psychologically affected’ by her ‘sister’s problem.’ As soon as her own diagnosis was confirmed, she applied for retirement. Unlike the male interviewees, early retirement did not entail a major shift in this woman’s identity, which gradually recentred around domestic work, distancing her from the image of an ‘invalid.’

*‘I worked, I used to drive, today I don’t drive because I have double vision. Today I stay at home and the work I do here is important too. I do everything around the house; the only thing I have help with is cleaning the bathroom, because it’s slippery, right? But I do everything, everything in my own time’ (MJD-1, woman, 46 years old).*

Listening to the accounts shared in association meetings and interviews, it became clear that men were more heavily impacted by compulsory retirement after diagnosis than women. For these men, having their careers abruptly cut short was yet another stigmatising aspect of the disease, while many of the women who had to leave their jobs were able to find alternative roles in domestic tasks and their family networks – roles socially recognised as ‘feminine’ and thus validated in their case. Although the women were constantly aware that, even in this sphere, a time would come when they had to ‘stop working’ too, it was experienced less dramatically since it tended to occur gradually as the disease progressed. For the men, by contrast, the sudden rupture from work was felt more keenly, associated with the expectation for them to be the family providers (even when their wives also worked outside the home). Above all, it meant their withdrawal from the public space in which they had constructed their relationships and their sense of belonging.

Gender expectations and the production of stigma also have a marked impact on the life experiences of those affected by Berardinelli Syndrome. Bodily stigma is a significant aspect of the social world of people living with BS and their families, shaping their lives – especially women’s – in various forms and intensities. The syndrome leads to pronounced thinness and muscular protrusions, particularly of the jaw, arms and abdomen, which can sometimes lead women to be mistaken for gay men or *travestis*^5^ and consequently stigmatised. However, the bodily experience of those living with BS is not reducible to suffering alone. There were also situations of exception, particularly among men affected by the condition.

As we were told, after a childhood marked by being ‘skinny,’ some male interlocutors developed muscular physiques as they entered adolescence and adulthood, which was seen as personally advantageous by many of them. In a Brazilian society that values the muscular male body cultivated in gyms, having such a physique affords these men inclusion in an aesthetically valued standard. One young man with BS, who stood out from others as someone who had enjoyed significant social inclusion since childhood, considered that his life had unfolded in what are perceived as ‘normal’ parameters.^6^ Nonetheless, he had also been through moments of ‘revelation’ of the illness in his intimate relationships with girlfriends. He explained that he never concealed his condition from the women he dated and indeed they encouraged him to take care of his health. At the same time, his account points to the fact that there was always a moment of disclosure or ‘revelation,’ which appears as a difficult episode, an ordeal through which someone seen as ‘discreditable’ – to use Goffman’s (1988) term – has to pass. This same young man also recounted the case of another male patient whose engagement was broken off when his fiancée learnt about his condition, which in his view underscored the ‘discrediting’ potentiality of the rare disease.

In the social lives of adult women with BS, contact with strangers in a range of situations and locations, including on the street, at school, in clinics, hospitals, supermarkets and leisure spaces, frequently entail embarrassment, ambiguity and discomfort (Goffman 1988) related to their physical appearance. Their corporeality carries a specific and unfavourable expressiveness that exposes them to forms of vulnerability and violence similar to those experienced by gay people, trans people and *travestis*. One of our interlocutors recounted more than one episode in which her daughter, on entering puberty, was mistaken for a gay man, a *travesti* or even a heterosexual man. In one situation, she had to defend her daughter from a group of boys who cornered her on the street, calling her a *viado*, a pejorative term for a gay man. However, one of the most discriminatory incidents occurred during a medical consultation when the healthcare professional categorically stated that her daughter ‘was a man.’

*‘He humiliated her so much! He called her a homosexual, and I kept telling him she wasn’t a man, but he responded: ‘She is a man!’ She was so ashamed that she hid under my arm! So I said to him: ‘If she were a man, she’d have a man’s name, she’s a woman. Look, if you don’t know about this condition she has, that’s your problem!’ I told him it was a syndrome and that there was already treatment for it, but he said: ‘You can tell me a hundred times and I still won’t believe this is a woman!’ And I said: ‘She is a woman, I gave birth to her, her name is a woman’s name, she is a woman!’’ (BSF-4).*

For young women with BS, these confrontations are often a persistent feature of social life due to the expectations placed on female corporeality. These are women whose muscular bodies can render them ambiguous to external perception, interpreted through cultural codes relating to sexual orientation and thus expected in turn to conform to a heterosexual norm and its corresponding physical indices. The social prejudices directed towards gay men and travestis permeate the lives of these women, despite the fact that they do not belong to either group. This illustrates the discriminatory and often violent effects of homophobia, which extend beyond those directly targeted and may also affect individuals who, intentionally or not, are perceived as physically resembling or being associated with them.

These experiences also illustrate how norms of bodily appearance and norms of gender and sexuality can operate together in the production of stigma. McRuer (2006) draws attention to the interconnection between bodily normativity and heteronormative expectations, showing how bodily difference can become entangled with assumptions about gender and sexuality. In the case of women with BS, their bodily characteristics are therefore not simply perceived as signs of physical difference, but may also be interpreted through gendered and heteronormative categories, producing ambiguity and stigma.

Once again, we also take inspiration from Goffman (1988) when he argues that although stigma is ‘a special kind of relationship between attribute and stereotype […] there are important attributes that almost everywhere in our society are discrediting’ (Goffman 1988: 7). While stigma clearly involves corporeality in interaction in the cases studied here, it also often produces moral readings. Indeed, this aspect affects women with BS in particular ways, their apparently masculinised physical appearance provoking moral accusations, such as being labelled gay or *travesti*. We can thus observe that there are diverse ways of dealing with the social stigma based on gendered corporeality in the case of people with BS. Because of their reduced body fat, women experience greater social vulnerability and stigmatisation than men with the same syndrome, as muscularity allows these men to perform gender and masculinity more easily.

In both research contexts, we can observe a continuous biographical process of re-signifying experiences of suffering, through which corporeality is reworked to align with more positive feelings that seek to transform *discrimination* into *empowerment*. In the search to give new meaning to this experience at a biographical level, no serious contradictions exist between the emotional and rational dimensions. On the contrary, the emotions involved in the lived experience of stigma are rationally organised in the development of coping strategies, among which associativism emerges as a path helping affected people to pursue their rights and recognition.

### ‘I’m not alone’: health activism as a way of confronting stigma

Families with a history of rare diseases (RDs) have engaged in political mobilisation on a global scale, exploring a range of initiatives with different emphases in pursuit of treatments and healthcare support (Akrich et al. 2013; Rabeharisoa et al. 2012, 2013; Rose 2007; Novas 2006; Rabinow 1999). In Brazil, associativism around RDs has helped ensure access to high-cost treatments, as well as increase the visibility of these conditions and the people affected by them, questioning the place assigned to patients as ‘victims of fate’ and instead asserting them as rights-bearing subjects (Aureliano and Gibbon 2020; Nóbrega 2020). In this process, social identities shaped by rare disease are reconfigured, along with the stigmas associated with them. ‘Not being alone anymore,’ ‘being part of a larger group’ and ‘finding a new family’ are recurrent expressions among our interlocutors who participate in patient associations and have found a collective form of constructing a positive identity in activism, working both with and beyond the medical condition:

*‘I think it’s good to see people coming together, because seeing one another gives us strength. When you don’t see anyone, you feel isolated, but when you look around and realise there are so many others in the same boat, you think, wow, I’m not alone’ (AR-1, woman, 62 years old).*

*‘There are people with other conditions worse off than we are, in terminal stages, and being here [at the association] is a way of showing that we’re alive, we still have so much to live for’ (MJD-1, woman, 46 years old).*

Virtual social networks have also allowed increased engagement among people living with rare diseases, especially those that cause disabilities affecting their mobility. In the case of the MJD patient association, some of the interactions among members gradually shifted to social media as the disease progressed in some of the founding members. This resulted in less regular in-person meetings but not the dissolution of the network established among them.

In the context of the research on people with BS, the creation of an association emerged from the encounter between two mothers, mediated by a paediatrician familiar with cases of children born with the so-called *doença dos magros* (‘disease of the thin’). He asked the women whether they knew any ‘people with the thin disease’ (*pessoas dos magros*), referencing the popular regional designation. After years spent searching for a diagnosis that could identify the cause of the condition and any forms of treatment, these two mothers founded the association and began publicly disseminating information about a rare disease in the region across small towns and settlements in the state’s interior, citing the medical terminology and providing guidance on how to access available treatments. Whenever they met new families who had relatives with the condition, the two association leaders engaged in a mediating and informative practice about BS (Nóbrega and Valle 2023).

*‘Since we had already received some guidance, we would say: look, your child needs to do some tests, because they have high [cholesterol] levels […] Over time, they were able to understand the problem and accept what we were saying […] We started teaching them the scientific name. Until then, they only knew it as the ‘disease of the thin’’ (BSF-2).*

It is also important to note that both women experienced profound forms of stigma in their roles as mothers, as they had to contend with discriminatory gazes and comments directed towards their children during childhood because of their distinctive bodily appearance. One of them told us about an episode that had deeply marked her. When her child was one month old, during one of their first public outings to a shopping centre, she was approached by a stranger. Looking at the baby, the woman asked: ‘*What is that*?’ and then went to remark that the child looked like ‘*a little monkey-boy.*’ After this episode, the mother was unable to care for – or even look at – her baby for some time:

*‘I spent seventeen days outside myself. They told me I had beaten the woman; I had torn all her clothes – until my husband arrived [to restrain me]. From that moment, I didn’t touch [my son] anymore, I didn’t look at him, didn’t breastfeed, didn’t change a single nappy, nothing! I had to go to my mother’s house […] In my subconscious, if I looked, I would see that little monkey in that child’ (BSF-3).*

The other mother leader in the social mobilisation of people with BS, experienced an analogous situation in her role as a teacher. She taught at the same school attended by her daughter, who has BS. She told us that on one occasion she was approached by one of her students, who referred to her daughter in a derogatory fashion:

*‘I was teaching a class when, out of nowhere, a boy said, ‘Miss, what about that daughter of yours who looks like a monkey?’ That day I totally lost it, you know? I didn’t say anything, I just walked out of the classroom. […] I only returned the next day. The next day the kids came to talk to me and I explained: ‘Look, my daughter might be ugly on the outside, but on the inside she’s not’’ (BSF-2).*

Diverging from the physical features expected of babies and infants, children with BS and their mothers may experience situations like these described above, in which they are left feeling humiliated and have their very humanity called into question by being associated with monkeys. The mothers also feel the stigma of being perceived as ‘mothers who failed’ to produce healthy and beautiful babies. Sometimes, they are also seen as negligent since their children’s thinness may also be misinterpreted as a sign of malnutrition.

The bond and mutual support that these two women developed between themselves and their families fuelled the initiative to found an association that could bring together a larger number of families. Gradually, the two mothers began to train as lay experts (Valle 2017; Epstein 1996) on the genetic nature of BS and established contact with the few geneticists working at the state’s public university where their children began to receive care at the university hospital. Through therapeutic and clinical relationships with doctors and health professionals, a kind of partnership was created that enabled the emergence and consolidation of their associativism.

The mothers who led the association of people with BS and their families used a range of strategies to get more people involved in their project of social mobilisation and the scientific and genetic divulgation of the disease. One of the strategies employed to make scientific information accessible to working-class families in the late 1990s was to distribute copies of a regional newspaper that had published a report on BS. To find people with the syndrome in the different municipalities in the Seridó region, the two mothers carried around photos of their own children and asked local residents whether they knew of any child who resembled the images. Through this routine practice of contacting other families with children and/or relatives with BS, more and more people came to be identified as living with BS in the region.

Following the creation of the association, demands for research, treatment and medication – along with social recognition able to counter stigmatisation – brought people with BS and their families into contact with medical geneticists, endocrinologists and other professionals in the scientific field. Through their routine clinical practice, these medical professionals instructed the families in accordance with a biomedical and genetic explanatory model. At the same time, the experience of collaborating in an association revealed the need for informational tools capable of supporting the demand for rights, justice and citizenship, which led to contact with actors possessing technical knowledge in the legal field. This involvement was especially clear in the work of the association’s founders, who would sometimes position themselves as ‘biosocial mediators and experts’ (Valle 2017) and who – due to their distinct social background compared to other families in this regional social context – had privileged access to medical specialists and public officials. This dimension was particularly evident in the judicialization of the right to health, which is implicated in the demands for access to leptin, but also in the struggle for social *respect* (Nóbrega and Valle 2023).

As noted above, collaboration between patient associations and medical specialists is a recurring feature of the rare disease field. In contexts across the Global North, such relationships have often fostered research initiatives aimed at developing cures or more effective therapies for affected individuals (Novas 2006). This is not the case in Brazil, where most patient associations operate under precarious economic and organisational conditions, relying largely on voluntary work and focusing primarily on raising awareness of the disease, promoting mutual support amongst affected individuals and their families, and facilitating access to available forms of care. In this context, the pursuit of a cure does not constitute the primary objective of most Brazilian associations. Rather, their efforts are directed towards securing the minimum standards of care to be provided by the state, while also strengthening families collectively in their efforts to confront the social challenges associated with rare disease.

In both analysed contexts, we can observe that the associations played a key role in confronting social stigma by seeking to foster feelings of collectivity and belonging, and by supporting the formation of new social identities grounded in genetic knowledge of their condition. Joining an association allowed families not only to share experiences and individual strategies for coping with the medical condition in everyday life, but also to equip themselves with medical information and explanations that could be deployed in situations where they were questioned about being intoxicated, for example, in the case of individuals with MJD, or about the unusual appearance of babies or women with BS. In both these studied cases, belonging to an association was described as like feeling part of a “large family,” with HRDs becoming seen as a biological connection between people who were not blood relatives but saw themselves as one big family, united by their shared rare condition and their common pursuit of rights and recognition.

## Conclusion

Stigma, like difference and identity, is a socially produced category, involving complex processes of negotiation shaped by power relations and regimes of truth that are continually contested in the formation of social identities associated with illness and other social markers. In our research, we were able to observe both the oppressive power of stigmatisation and the strategic deployment of stigma within social mobilisation or political activism, which transform stigmatisation into a tool for contesting the same forces that produce it by shifting away from the notion of “victims of a disease” to “citizens with rights”.

In line with analyses that emphasise the structural dimensions of stigma (Thomas et al. 2025; Charmaz 2020; Hansen et al. 2014), we identified, in both cases examined, the operation of processes of social hierarchisation and the persistence of historical forms of moral delegitimation, evident in the reproduction of gender relations as well as in the meanings attached to non-heteronormative sexual identities. These structural dimensions become apparent through experiences that resonate with those of groups who share certain social similarities. This was particularly evident amongst women living with BS, who were frequently mistaken for gay men or travestis. In a different register, non-conformity with gendered expectations—such as the ideal of the male breadwinner or the female caregiver—may itself generate stigmatising experiences as the disease becomes visible. This was observed both amongst men living with MJD and amongst mothers of children with BS, who were at times perceived as neglectful because of their children’s extreme thinness.

The understanding of stigma emerging from the contexts analysed here attests to the relational dimensions of hereditary rare diseases (HRDs), both in genetic and social terms. This invites reflection on how families and communities may themselves become marked by a hereditary rare disease when, for instance, the condition ceases to be perceived as “rare” within a particular local context, such was the case of BS, which acquired a local designation in the Seridó region due to the concentration of cases identified in that territory. By contrast, stigmas associated with disability generated dilemmas concerning the disclosure of a family diagnosis of MJD in workplaces and in everyday social relationships. The hereditary character of these conditions highlights that stigma often exceeds the individual body, extending across kinship networks and community relations.

Although the suffering associated with stigmatisation was present in many of our interlocutors’ narratives, we found that contact with and participation in patient associations provided patients and their families with new repertoires through which to speak about themselves and their condition in social encounters. Medical terminology and genetic knowledge were mobilised to challenge moralising interpretations of their illness and to contest stigmatising readings associated with bodily expectations that emerged in these interactions, becoming a resource for the production of collective identities, claims-making, and social recognition. Such knowledge was not deployed solely in everyday encounters with lay people; it was also mobilised in clinical interactions with health professionals unfamiliar with rare diseases, a recurring theme in our interlocutors’ accounts. In this respect, alliances with geneticists and other specialists, facilitated through patient associations, were viewed as a means of promoting forms of health education that should also be directed towards medical professionals more broadly. Physicians themselves form part of the institutional structures through which discourses of normality, embodiment, gender, and health may contribute to the production and reproduction of social stigma.

We therefore argue that the production of visibility around rare diseases, as demanded by affected individuals and their families, has become an important mechanism for confronting stigma not only at the individual level, but also within families, communities and institutions. In this regard, efforts to secure recognition and health rights by people living with rare diseases share important affinities with other social movements that emerged throughout the twentieth and twenty-first centuries, including the disability movement (Barnes and Mercer 2010) and HIV/AIDS activism (Epstein 1996). What these movements share is a challenge to dominant norms surrounding bodies, gender, and sexuality. Although their struggles are not identical in all respects, they converge in questioning the assumptions underlying compulsory bodily normativity (McRuer 2006).

By bringing together two different groups of people living with hereditary rare diseases, our aim was to identify common elements across their experiences, including the search for lay explanations of illness, the challenges encountered in everyday social interactions, and collective strategies for confronting stigma. We argue that placing these two ethnographic fields in dialogue sheds light on the ways in which longstanding social categories continue to shape experiences of stigma amongst people living with HRDs, while also revealing efforts to displace the image of the “different body” towards a broader field of possibilities concerning what it means “to be normal” and, ultimately, what it means to be human.

Further research is needed to expand this comparative framework and to examine additional social markers that also structure the experiences of people living with HRDs. Race and class, for example, warrant particular attention, as they play a central role in the production of social inequalities and, consequently, in the structural experience of stigma. Longitudinal studies involving multiple generations of families living with HRDs are therefore essential for understanding how stigma and the mechanisms through which it is challenged evolve across generations, as well as how everyday experiences of stigmatisation are shaped by broader institutional arrangements, public policies, biomedical discourses, and social inequalities.

## Data Availability

No datasets were generated or analysed during the current study.

## Footnotes

1 Lipodystrophies are a broad group of disorders affecting adipose tissue and characterised by a reduction of fat in different parts of the body.

2 In anthropology, the term ‘interlocutor’ refers to the person with whom the anthropologist dialogues and participates in the research process, recognizing that this person is an active subject in the construction of knowledge and cultural interpretation. The term highlights the dialogical nature of ethnographic research, which emphasizes active participation, dialogue and the joint production of knowledge between the researcher and the individuals under study.

3 To consult the approval on Ethics Committees, access https://plataformabrasil.saude.gov.br/login.jsf. Numbers of protocol (CAAE): 27146514.5.0000.5257 and 85587618.1.0000.5537.

4 Leptin is a hormone naturally produced by the human body, but in people with BS it is not produced due to lipodystrophy. The medication developed to replace leptin in patients with BS is Myalept, manufactured by Aegerion Pharmaceuticals.

5 *Travesti* is a gender identity recognised in Brazil and other parts of Latin America, referring to individuals assigned male at birth who live and express themselves in the feminine gender, while not necessarily identifying as women or as transgender women. Because of its specific historical, cultural, and political meanings, the term is retained in Portuguese throughout this article.

6 Attending school, working, seeing friends and going to parties were mentioned as routines associated with living a ‘normal life.’
